# Tpl2 Ablation Leads to Hypercytokinemia and Excessive Cellular Infiltration to the Lungs During Late Stages of Influenza Infection

**DOI:** 10.3389/fimmu.2021.738490

**Published:** 2021-10-07

**Authors:** Krishna Latha, Katelyn F. Jamison, Wendy T. Watford

**Affiliations:** ^1^Department of Infectious Diseases, University of Georgia, Athens, GA, United States; ^2^Department of Cellular Biology, University of Georgia, Athens, GA, United States

**Keywords:** Tpl2, MAP3K8, influenza, interferons, inflammatory monocytes, neutrophils, CCL2

## Abstract

Tumor progression locus 2 (Tpl2) is a serine-threonine kinase known to promote inflammation in response to various pathogen-associated molecular patterns (PAMPs), inflammatory cytokines and G-protein-coupled receptors and consequently aids in host resistance to pathogens. We have recently shown that *Tpl2^-/-^* mice succumb to infection with a low-pathogenicity strain of influenza (x31, H3N2) by an unknown mechanism. In this study, we sought to characterize the cytokine and immune cell profile of influenza-infected *Tpl2^-/-^* mice to gain insight into its host protective effects. Although *Tpl2^-/-^* mice display modestly impaired viral control, no virus was observed in the lungs of *Tpl2^-/-^* mice on the day of peak morbidity and mortality suggesting that morbidity is not due to virus cytopathic effects but rather to an overactive antiviral immune response. Indeed, increased levels of interferon-β (IFN-β), the IFN-inducible monocyte chemoattractant protein-1 (MCP-1, CCL2), Macrophage inflammatory protein 1 alpha (MIP-1α; CCL3), MIP-1β (CCL4), RANTES (CCL5), IP-10 (CXCL10) and Interferon-γ (IFN-γ) was observed in the lungs of influenza-infected *Tpl2^-/-^* mice at 7 days post infection (dpi). Elevated cytokine and chemokines were accompanied by increased infiltration of the lungs with inflammatory monocytes and neutrophils. Additionally, we noted that increased IFN-β correlated with increased CCL2, CXCL1 and nitric oxide synthase (NOS2) expression in the lungs, which has been associated with severe influenza infections. Bone marrow chimeras with Tpl2 ablation localized to radioresistant cells confirmed that Tpl2 functions, at least in part, within radioresistant cells to limit pro-inflammatory response to viral infection. Collectively, this study suggests that Tpl2 tempers inflammation during influenza infection by constraining the production of interferons and chemokines which are known to promote the recruitment of detrimental inflammatory monocytes and neutrophils.

## Introduction

Seasonal influenza A virus (IAV) infections account for approximately $11.2 billion in total economic burden to the healthcare system (1), 500,000 hospitalizations and 40,000 deaths per year (2). While vaccination does prevent severe disease, the efficacy of each seasonal vaccine is variable. Factors such as the inaccurate prediction of the seasonal strains, poor immunogenicity of the vaccination, vaccine production issues and public vaccination non-compliance all contribute to suboptimal influenza vaccine efficacy each season (3). Treatment options for influenza are adamantane drugs that inhibit the matrix protein 2 (M2) ion channel and inhibitors that target the neuraminidase surface protein (4). However, with the high rate of viral mutation, rapid development of resistance to these antivirals has been observed, with approximately 45% of IAV strains worldwide already resistant to adamantanes as of 2013 (5) and growing resistance against neuraminidase inhibitors (6). Despite these available interventions, influenza infections still account for 3.4% of critical illness hospitalizations, even during moderate seasons (7).

Many factors contribute to influenza-associated hospitalizations and deaths, such as underlying medical problems, secondary bacterial pneumonia and congestive heart failure. A common feature of severe disease progression in many patients with such comorbidities is hypercytokinemia, the over-production of soluble host-derived pro-inflammatory mediators initially intended to restrict local virus spread but whose dysregulation leads to systemic inflammation and potentially life-threatening complications (8). Hypercytokinemia is more prevalent in cases of avian influenza or lethal pandemics (9, 10) compared to seasonal influenza, correlating the higher cytokine levels with severe disease progression (11–13).

Cytokines are secreted in response to influenza infection of target cells that possess cellular sensors for viral components. For example, RIG-I-like receptors (RLRs) and Toll-like receptors (TLRs) recognize viral genomic RNA and initiate a signaling cascade, *via* NF-κB, MAPKs and interferon regulatory factors (IRFs), that leads to the production of pro-inflammatory cytokines and interferons (IFNs). These, in turn, induce an anti-viral response in neighboring cells to limit viral spread. Complex signaling networks further lead to cytokine release by neighboring cells (14). Proinflammatory cytokines and chemokines direct the rapid recruitment of innate immune cells, comprising neutrophils, natural killer (NK) cells, and inflammatory monocytes (15) which home to the site of infection. CXCL1 promotes neutrophil recruitment, whereas CCL2 recruits inflammatory monocytes (16, 17). Neutrophils and inflammatory monocytes are responsible for early viral control, but their dysregulation can also inadvertently damage host tissues and cause severe immunopathology systemically.

In cases of severe influenza disease, as with highly pathogenic avian influenza (HPAI), hypercytokinemia promotes excessive recruitment of neutrophils and inflammatory monocytes through overproduction of IFNs, IL-6, IL-1β, CCL2, CCL3, TNF, and IP-10 (10, 12, 13, 18). These cells have been shown to contribute to pathology through the expression of effector molecules that promote viral clearance but also contribute of host immunopathology, including inducible nitric oxide synthase (iNOS), myeloperoxidase (MPO) and TNF-α Related Apoptosis Inducing Ligand (TRAIL). Because of potentially deadly consequences of hypercytokinemia during influenza and other viral infections, it is critical that we gain a better understanding of its regulation including factors that shift the balance from a beneficial towards a pathological response and *vice versa*. These molecules represent potential therapeutic targets for patients with known comorbidities or those who otherwise develop severe influenza disease.

Tumor progression locus 2 (Tpl2, also known as COT or MAP3K8) has been shown to regulate the immune response to a variety of intracellular pathogens including *T. gondii*, *L. monocytogenes*, *M. tuberculosis*, and influenza virus (19–22). Tpl2 is a serine-threonine kinase that is expressed in various cell types with functions that differ depending on cell type and stimulus (22–24). Tpl2 is most widely recognized for its role in the mitogen-activated protein kinase (MAPK) pathway. For example, Tpl2 transmits signals downstream of TLRs and RLRs *via* activation of MEK1/2, ERK1/2, and p38 (25–27). Tpl2 exists in an inactive complex with p105 and ABIN2 (28). Upon activation by IKK phosphorylation, p105 is proteolyzed to p50 dimers, releasing Tpl2 and enabling it to initiate downstream signaling of the ERK pathway (23, 29, 30). Consequently, Tpl2 promotes the expression of pro-inflammatory cytokines such as IL-6 (31), TNF (23, 32) and IL-1β (33) and constrains the expression of IL-12 (19) and type I IFNs (20, 21, 34). Notably, these Tpl2-regulated cytokines have been implicated in severe influenza disease. We previously demonstrated that *Tpl2^-/-^* mice show more severe disease in response to low pathogenicity influenza infection and succumb to infection by 10 days post infection (dpi) (22). In this study, we sought to characterize the cytokine and immune cell profile of influenza-infected *Tpl2^-/-^* mice to gain insight into its host protective effects.

Despite modestly increased viral titers in the *Tpl2^-/-^* mice throughout the course of infection, the titers consistently decreased over time to undetectable levels by 9 dpi, confirming that impaired viral clearance failed to explain the observed mortality in *Tpl2^-/-^* mice. Instead, we demonstrate that Tpl2 ablation disrupts the balance between beneficial and pathologic immune cell activity, leading to excessive accumulation of inflammatory monocytes, neutrophils, and NOS2 production in lungs of *Tpl2^-/-^* mice by 7 dpi. This imbalance is attributed to an excessive type I IFN signature observed in the lung tissue of *Tpl2^-/-^* mice late in the course of infection. Tpl2 deficiency partly recapitulates the severe immunopathology observed in human HPAI infections, including excessive monocyte and neutrophil recruitment. Therefore, understanding how Tpl2 regulates IFN-β and downstream late-stage responses to influenza may lead to better interventions for viral-induced lung immunopathology.

## Materials and Methods

### Ethics Statement

All animal experiments were performed in accordance to the national guidelines provided by “The Guide for Care and Use of Laboratory Animals” and the University of Georgia Institutional Animal Care and Use Committee (IACUC). The IACUC approved all animal experiments.

### Mice and Viruses

Wild type (WT) C57BL/6 mice were purchased from the Jackson Laboratory. *Tpl2^-/-^* mice backcrossed 10 generations onto the C57BL/6 strain were kindly provided by Dr. Philip Tsichlis (32). Animals were housed in micro-isolator cages in the Coverdell Rodent Vivarium, UGA. Both male and female mice were used in experiments to evaluate sex as a biological variable.

Embryonated, specific pathogen-free chicken eggs were purchased from Poultry Diagnostics and Research Center, UGA. Influenza virus A/HKx31 (H3N2; hereafter x31) stocks were propagated in the allantoic cavity of 9- to 11-day-old, embryonated, specific pathogen-free (SPF) chicken eggs at 37°C for 72 hours, and viral titers were enumerated by plaque assays. Madin Darby Canine Kidney (MDCK) cells were cultured and plated on a 12-well plate at a concentration of 5x10^5^ cells/well. After 24 hours the well is generally confluent, and 100 μl of serially diluted sample was added to the well, along with 200 μl of the Infection Media (Minimal Essential Media containing 1 µg/ml TPCK treated typsin and lacking serum). The sample was allowed to incubate with the cells for an hour at 37°C to promote infection of the monolayer, followed by the addition of 2.4% Avicel in Overlay Media (Infection Media with 40 mM HEPES, 4 mM L-gutamine, 200 U/ml penicillin, 200 U/ml streptomycin and 0.15% Sodium Bicarbonate) to facilitate localized viral infection and plaque formation. After 72 hours, wells were washed with PBS, cells were fixed with 60% acetone:40% methanol, and plaques were stained with crystal violet (made by mixing one volume of 0.0012 w/v of crystal violet powder in 5% Methanol, 11.1% Formaldehyde, 60% H_2_O with one volume of PBS) for visualization and enumeration.

### Influenza Infection of Mice

Age-matched, 6- to 8-week-old, WT and *Tpl2^-/-^* mice were anesthetized with approximately 250 mg/kg of 2% weight/volume Avertin (2,2,2- Tribromoethanol, Sigma) followed by intranasal instillation of 50 µl PBS containing 10^4^ pfu of influenza A/HKX31 (H3N2, hereafter referred to as x31). The mice were studied for their susceptibility to infection by measuring daily weight loss and clinical scores according to the following index: piloerection, 1 point; hunched posture, 2 points; rapid breathing, 3 points. Mice with a cumulative score of 5 or that had lost 30% of their initial weight were humanely euthanized.

### Tissue Collection

Mice were sacrificed at 7 to 9 dpi. Blood was collected from the heart by cardiac puncture into serum collection tubes, centrifuged at 9000 x *g* for 5 min, and the sera were stored at -80°C until cytokine analysis. Bronchoalveolar lavage fluid (BALF) was obtained from the lungs prior to harvest using 1 ml of PBS instilled twice into the lungs. The BALF was centrifuged at 500 x *g* for 5 min, and the cell-free BALF was stored at -80°C until cytokine analysis; the cellular pellet was lysed in TRK lysis buffer (E.Z.N.A Omega Bio-Tek, Inc. Norcross, GA, USA) for quantitation of gene expression. The lungs were perfused with 10 ml of PBS injected directly into the right ventricle of the heart. Lungs were harvested into 1 ml of PBS and homogenized in a bead mill homogenizer (Qiagen Tissue Lyser II) at 25 hz for 2-4 min. The homogenate was centrifuged at 500 x *g* for 5 min, and the pre-cleared homogenate was either: (1) directly aliquoted for viral titer assessment, (2) lysed in TRK tissue lysis buffer for RNA extraction, or (3) centrifuged at 5000 x *g* for 5 min to clarify the homogenate for cytokine analysis by ELISA. For mice sacrificed at 9 dpi, whole lungs were processed without perfusion or BALF harvesting.

### Cytokine Analysis

Cytokine quantitation in the blood, BALF, and clarified lung homogenates was performed using the Mouse Inflammation Cytometric Bead Array (CBA) (IL-6, IFN-γ, MCP-1, TNF, IL-10 and IL-12p70, Becton Dickenson), Mouse ProcartaPlex 9-plex (RANTES, IP-10, MIP-1α, MIP-1β, IL-1α, IL-1β, IL-28, GM-CSF, Invitrogen), Standard murine ABTS ELISA Development kit (CXCL1 & IFN-γ, Peprotech) and Lumikine express kits (IFN-β, Invivogen).

### Cellular Analysis

At 4 and 7 dpi, the following protocol was used to assess the BALF and lung cellular composition after the BALF harvesting and lung perfusion as noted above. The lungs were harvested into Hyclone RPMI media (15-040-CV, Corning, Manassas, VA) containing 10% FBS and 2 mM L-glutamine (Invitrogen, Grand Island, NY). Lungs were minced with razor blades, and incubated in EDTA solution [RPMI 1640 containing 0.01 M HEPES (Lonza, Walkersville, MD), 1.25 mM EDTA (Fisher Bioreagents, Fair Lawn, NJ),] for 1 hour at 37°C in an incubator shaking at 250 RPM. The tissue was centrifuged at 350 x *g* for 10 min and then digested with 10 mL of collagenase solution [RPMI 1640 containing 1 mM CaCl_2,_ 0.01 M HEPES (Lonza, Walkersville, MD), 2 mM L-glutamine (Invitrogen, Grand Island), 100 U/ml penicillin, 100 U/ml streptomycin, 5% FBS, 0.2 µg/mL Gentamicin, and 150 U/ml collagenase (Sigma-Aldrich C2139)] for 30 min at 37°C in an incubator shaking at 350 RPM. The digested tissue was passed through a 70 µm cell strainer, and the cell suspension was centrifuged at 350 x *g* for 10 min, resuspended in 44% Percoll, and layered on top of 67% Percoll. The gradients were spun at 900 x *g* for 20 min (without brake), and the enriched leukocytes were recovered from the interface. The cells were washed with PBS at 350 x *g* for 10 min and enumerated using an automated cell counter (Cell Countess, Life Technologies).

Cells were stained at 4°C for 20 min with fluorescently-labeled antibodies against the following cell surface markers in the presence of Fc blocker (eBioscience, San Diego, CA location): Siglec F, CD11b, CD11c, Ly6C, Ly6G, CD45.2 (Stain 1); TCRαβ, TCRγδ, CD4, CD8, DX5, CD45.2 (Stain 2). The cells were fixed with 1% formalin and analyzed on the LSR II flow cytometer (BD Biosciences). CD45.2-gated hematopoietic-derived leukocyte populations were characterized as follows: inflammatory monocytes (Siglec F^-^, CD11b^high^, CD11c^low^, Ly6C^+^), neutrophils (Siglec F^-^, CD11b^high^, CD11c^low^, Ly6G^+^), alveolar macrophages (Siglec F^high^, CD11b^int^), eosinophils (Siglec F^high^, CD11b^high^), NK cells (αβTCR^-^, DX5^+^), CD4 T cells (αβTCR^+^, CD4^+^), CD8 T cells (αβTCR^+^, CD8^+^), and γδ T cells (αβ TCR^-^, γδ TCR^+^). The gating strategy is shown in **Supplementary Figure 3**.

### Analysis of Gene Expression

Messenger RNA was extracted from lung homogenates using the E.Z.N.A. Total RNA kit (Omega Bio-Tek, Inc. Norcross, GA, USA) and converted into cDNA using a High Capacity RNA-to-cDNA kit (Thermo Fisher, Waltham, MA) according to the manufacturer’s protocol. Relative expression of various genes was assessed using Sensifast Probe Hi-ROX kit (BIO-82020 Bioline, Taunton, MA) and probes sourced from Applied Biosystems (Beverly, MA) using a StepOne Plus instrument (Applied Biosystems, Beverly, MA). Results are expressed relative to the actin internal control and the WT or untreated sample using the ΔΔC_T_ method. The probes used are as follows: IFNβ1(Mm00439552), IFNα1(Mm03030145), IFNα4(Mm00833969), IFNγ(Mm0116813), IL-6(Mm00446190), IL1β(Mm00434228), CCL2(Mm00441242), CXCL1(Mm04207460), CCL5(Mm01302427), TNFSF10(Mm01283606), NOS2(Mm00440502), MPO(Mm01298424), STAT1(Mm00439518), SOCS1(Mm01342740), IL-10(Mm01288386), SOCS3(Mm01249143), SOCS4(Mm00439518) and STAT4(Mm00448890)

### Chimera Experiments

WT and *Tpl2^-/-^* mice were irradiated at 1100 Rads after reaching adulthood (> 6 weeks of age) and then injected with bone marrow from C57BL/6 mice at 3 x 10^6^ cells in 200 µl PBS. The mice were then maintained on acidified water (pH 2.5) for 2 months to allow for reconstitution of the hematopoietic compartment with WT cells. The resulting chimeras were infected with 10^4^ pfu x31 virus, and body weights were measured over a period of 8 to 10 days, at which times the mice were euthanized to assess the cytokine profiles on days with varying pathologies.

### Statistical Analysis

*P* values were calculated with GraphPad PRISM software version 9.2.0(332) using (one-way ANOVA with Tukey’s multiple comparisons test. Differences were considered statistically significant if *p* ≤ 0.05. Data represent means ± SEM. Survival data are graphed as Kaplan-Meier plots using GraphPad PRISM software, and *p* values were determined by Mantel-Cox test. Gaussian Correlation was performed to calculate the coefficient based on Pearson’s Correlation test with a two-tailed test. Simple Linear Regression analysis was also performed to analyze the best fit value or the slope and intercept to see if the two variables being compared for a particular genotype correlated or not (as seen by the straight dashed line). Additionally, the confidence interval was set at 95% (as seen by the curved dashed line).

## Results

### Tpl2^-/-^ Mice Succumb to Influenza Approximately 9 Days Post Infection

**Figure 1:**
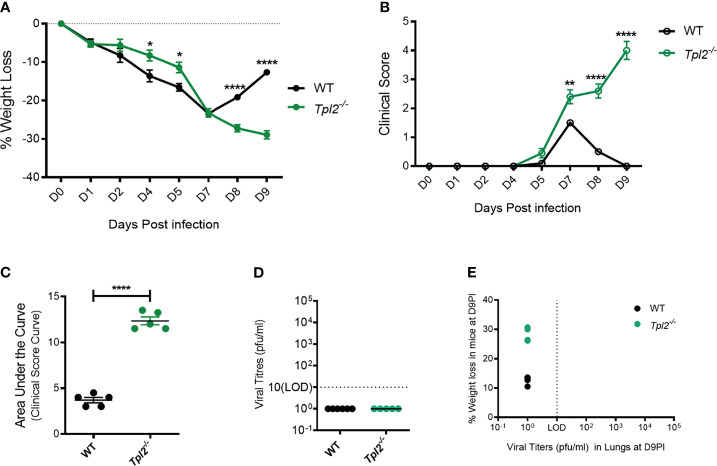
Severe pathology in influenza-infected *Tpl2^-/-^* mice does not correlate with viral load. **(A)** Percent weight change of WT (n = 5) *versus Tpl2^-/-^* (n = 5) mice 9 dpi with 10^4^ pfu influenza A virus strain x31. Data are representative of 5 experiments Unpaired student’s *t*-test; *p<0.05, **p<0.01, ****p<0.0001. **(B)** Progression of clinical symptoms including lethargy, piloerection, and hunching is shown throughout the course of infection. Data are representative of 5 experiments. Unpaired student’s *t*-test; *p<0.05, **p<0.01, ****p<0.0001. **(C)** Area under the curve statistics. Data are representative of 5 experiments, Area Under the Curve analysis was performed per genotype in Prism and then compared statistically by Unpaired student’s *t*-test; *p<0.05, **p<0.01, ****p<0.0001. **(D)** Lung viral titers (pfu/ml) were quantitated at 9 dpi. Baseline represents the limit of detection (LOD = 10 pfu/ml). Undetectable virus loads were assigned a value of 1. **(E)** Correlation of viral titers with weight loss at 9 dpi. Data are representative of 2 experiments. Two tailed Pearson’s Correlation test was performed *p<0.05, **p<0.01.

We have previously observed that *Tpl2^-/-^* mice are more susceptible than WT mice to influenza A virus infection using a low pathogenicity strain (x31; H3N2) (22). We reasoned that increased morbidity in the *Tpl2^-/-^* mice was likely due to impaired or delayed viral clearance or to an excessive anti-viral immune response. To distinguish between these possibilities, we infected WT and *Tpl2^-/-^* mice with x31 and monitored viral titers and inflammatory cytokine production as functions of morbidity and mortality late during the disease course. WT mice showed signs of disease from 1 to 7 dpi, at which time they began to recover as evidenced by weight gain and decreasing clinical scores (**Figures 1A–C**). In contrast, *Tpl2^-/-^* mice displayed progressive weight loss and increasing clinical symptoms from 7 to 9 dpi (**Figures 1A–C**). Notably, the weight loss and clinical symptoms were not different between male and female mice after 7 dpi with influenza (**Supplementary Figures 1A–D**). Importantly, despite severe clinical symptoms in *Tpl2^-/-^* mice, no virus was observed in either WT or *Tpl2^-/-^* mice at peak morbidity and mortality (9 dpi; **Figure 1D**), demonstrating that both strains had successfully cleared the virus by this time point. As expected from these findings, there was no correlation between morbidity as measured by weight loss and viral titers (**Figure 1E**), confirming that the morbidity observed in influenza-infected *Tpl2^-/-^* mice was not due to increased viral loads.

**Figure 2:**
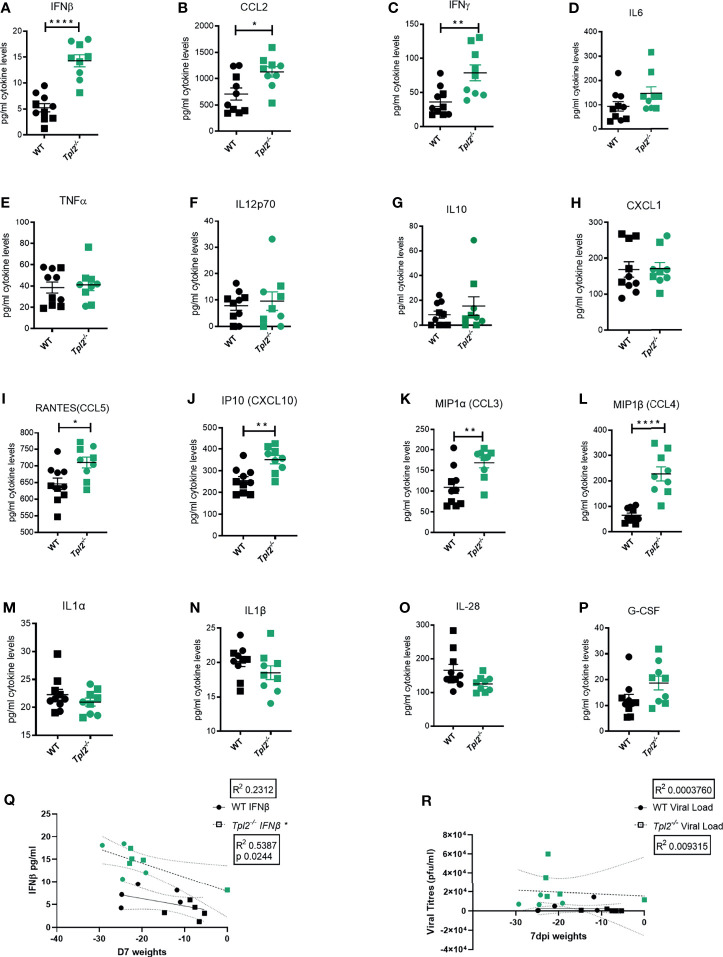
Excessive IFN cytokine signature is observed in influenza-infected *Tpl2^-/-^* mice at 7 dpi. WT (n = 10) and *Tpl2^-/-^* (n = 9) mice were infected intranasally with 10^4^ pfu of influenza x31 and euthanized at 7 dpi. **(A–P)** The lungs were homogenized for analysis of cytokine expression. Squares represent male mice, and circles represent female mice. Data are representative of 2 experiments. Unpaired student’s *t*-test *p<0.05, **p<0.01, ****p<0.0001. **(Q)** Correlation of Interferon levels in the perfused and lavaged lungs with weight loss at 7 dpi. **(R)** Correlation of viral titers in the perfused and lavaged lungs with weight loss at 7 dpi. Data are representative of 2 experiments. Two tailed Pearson’s Correlation test was performed *p<0.05.

Hypercytokinemia is widely reported in severe influenza-infected patients that eventually succumb to disease (9, 13). It is characterized by significantly increased levels of interferons (IFNs), IL-6, TNF, IL-12, IL-1β and various chemokines such as CCL2, MIP-1α, MIP-1β, RANTES, and IP-10 (9, 13). Therefore, the pro-inflammatory cytokine profile of lung homogenates from influenza-infected WT or *Tpl2^-/-^* mice was assessed using a multiplex protein assay. Significantly increased levels of IFN-β, CCL2, IFN-γ, CCL3, CCL4, CCL5 and CXCL10 were observed in the lung tissue of influenza-infected *Tpl2^-/-^* mice compared to WT (**Figures 2A–C, I–L**); increased levels of IFN-β, IL-6 and IL-10 were observed in the air spaces of *Tpl2^-/-^* mice (**Supplementary Figures 2H, K, N**); and increased levels of IFN-γ were observed in the blood of *Tpl2^-/-^* mice at 7 dpi (**Supplementary Figure 2C**). Notably, the levels of some cytokines associated with influenza infection were unaltered (**Figures 2D–H, M–P**) and **Supplementary Figures 2A, B, D–G, I, J, L, M**). These data demonstrate that *Tpl2^-/-^* mice display increased levels of pro-inflammatory cytokines and chemokines typically observed in human patients with influenza-induced hypercytokinemia (8–11). Notably, increased weight loss at 7 dpi correlated with high levels of IFN-β in the lungs of the *Tpl2^-/-^* mice (**Figure 2Q**). However, there was no correlation between weight loss and viral load in the tissue at 7 dpi (**Figure 2R**), as was the case at 9 dpi (**Figure 1E**). These data demonstrate that the morbidity in *Tpl2^-/-^* mice is due to the overexuberant immune response in *Tpl2^-/-^* mice at the late stage of influenza infection rather than impaired viral control.

### Tpl2^-/-^ Mice Are Characterized by Excessive Inflammatory Infiltration of the Lungs at 7 Days Post Influenza Infection

**Figure 3:**
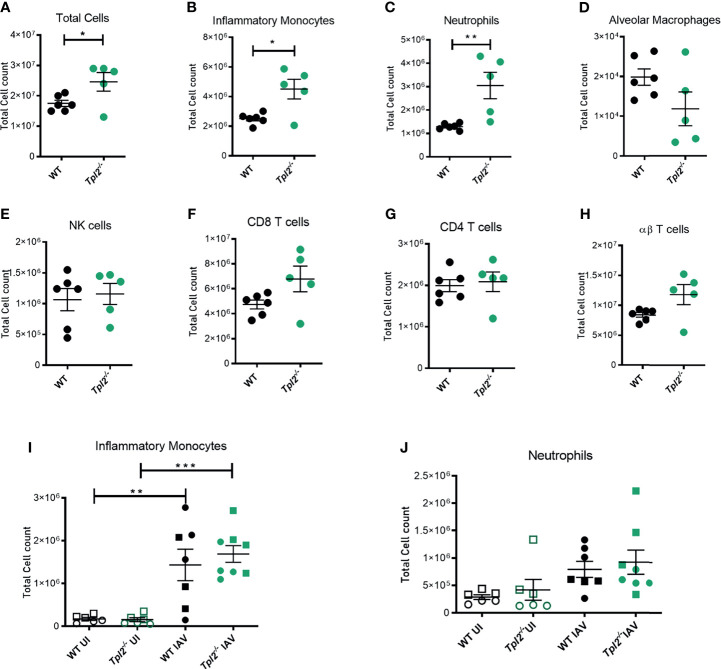
Excessive cellular influx of inflammatory monocytes and neutrophils in *Tpl2^-/-^* mice infected with influenza. WT and *Tpl2^-/-^* mice were infected intranasally with 10^4^ pfu of influenza x31 and euthanized at 7 dpi. The lungs were lavaged, perfused with PBS, digested with collagenase, and interstitial leukocytes were enriched by Percoll density gradient centrifugation **(A–H)**. Cell populations of infected WT (n = 6) and *Tpl2^-/-^* (n = 5) mouse lungs (post lavage, perfusion and digest) at 7 dpi are shown. Data are representative of 3 experiments. **(I, J)** Infiltrating cell populations were also assessed for WT (n = 7) and *Tpl2^-/-^* (n = 8) mice at 4 dpi (post lavage, perfusion and digest), including uninfected controls (UI). Data are representative of 3 experiments. Unpaired student’s *t*-test *p<0.05, **p<0.01, ***p<0.001. Squares represent male mice, and circles represent female mice.

We next assessed the cellular composition of the lung tissue and alveolar air spaces to identify cells that would be consequently recruited due to the hypercytokinemia and could contribute to tissue damage in the lungs and mortality. Mice were infected with influenza, and bronchoalveolar lavage fluid (BALF) and lung tissue were harvested at 7 dpi for analysis of cellular composition by flow cytometry (**Supplementary Figure 3**). The total cellularity of the lungs was significantly increased in *Tpl2^-/-^* mice. Higher numbers of cells were present in the perfused and lavaged lung tissue of the *Tpl2^-/-^* mice (**Figure 3A**). Furthermore, *Tpl2^-/-^* mice had significantly increased absolute numbers of inflammatory monocytes and neutrophils compared to WT mice at 7 dpi (**Figures 3B, C**). An increase in frequency of inflammatory monocytes and neutrophils was also noted (**Supplementary Figures 4A, B**), however no differences in total alveolar macrophages, NK cells, CD4 or CD8 αβ T cells or even γδ T cells were observed (**Figures 3D–H**). Therefore, it is the numerical increase in inflammatory monocytes and neutrophils that account for the higher total cellular infiltrates observed in infected *Tpl2^-/-^* mice. Typically, innate immune cells are recruited early during influenza infection to phagocytose or endocytose infected cells (35); therefore, it was unexpected to see such high numbers of them late in the infection in *Tpl2^-/-^* mice. Because Tpl2 deficiency has been demonstrated to impair monocyte, macrophage and neutrophil recruitment in response to inflammatory stimuli (21, 36–39), we further assessed the kinetics for the paradoxically increased monocytes and neutrophils in the lung tissue of influenza-infected *Tpl2^-/-^* mice. Therefore, we characterized the cellular composition of lungs at an earlier time point (4 dpi) with uninfected mice as negative controls. Although we noted influenza infection-induced recruitment of both inflammatory monocytes and neutrophils by 4 dpi, there was no difference between the WT and *Tpl2^-/-^* mice (**Figures 3I, J**). This was also true of all the other cell types examined (**Supplementary Figures 4E, F**). These data suggest a late acting effect of Tpl2, possibly in limiting the amplitude of the response or in promoting resolution of inflammation.

### NOS2 Is Overexpressed in the Lungs of Influenza-Infected Tpl2^-/-^ Mice

**Figure 4:**
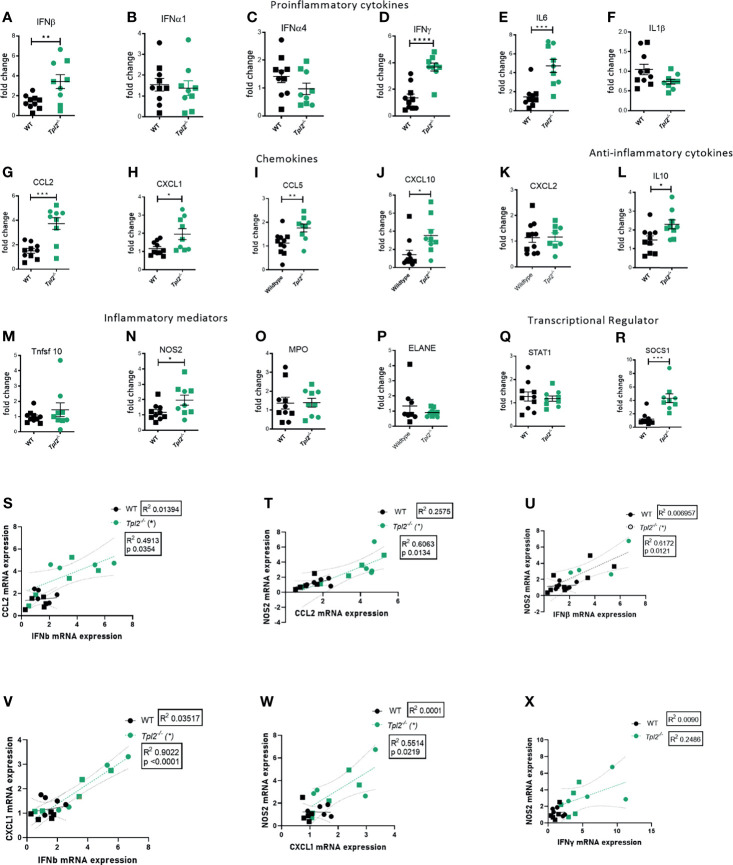
Increased mRNA expression of proinflammatory mediators in lungs of influenza-infected *Tpl2^-/-^* mice at 7 dpi. **(A–R)** WT (n = 10) and *Tpl2^-/-^* (n = 9) mice were infected intranasally with 10^4^ pfu of influenza x31 and euthanized at 7 dpi. The lungs were homogenized, and RNA was extracted and analyzed for gene expression by real-time PCR for pro-inflammatory cytokines **(A–F)**, chemokines **(G–K)**, anti-inflammatory cytokine **(L)**, inflammatory mediators **(M–P)** and transcriptional regulators **(Q, R)**. Unpaired student’s *t*-test; *p<0.05, **p<0.01, ***p<0.001, ****p<0.0001). **(S)** Pearson’s correlation of CCL2 mRNA *versus* IFN-β mRNA on day 7. *p<0.05 **(T)** Pearson’s correlation of NOS2 mRNA *versus* CCL2 mRNA on day 7. *p<0.05 **(U)** Pearson’s correlation of NOS2 mRNA *versus* IFN-β mRNA on day 7. *p<0.05 **(V)** Pearson’s correlation of CXCL1 mRNA *versus* IFNβ mRNA on day 7. *p<0.05 **(W)** Pearson’s correlation of NOS2 mRNA *versus* CXCL1 mRNA on day 7. *p<0.05 **(X)** Pearson’s correlation of NOS2 mRNA *versus* IFN-γ mRNA on day 7. *p<0.05 Data are representative of 2 experiments. Squares represent male mice, and circles represent female mice.

Highly pathogenic influenza viruses induce exaggerated immune responses that cause immunopathology *via* damage to the pulmonary epithelium by the recruited immune cells and their effector molecules (17, 40). A study of juvenile mice that exhibit severe disease in response to influenza infection revealed recruitment of inflammatory monocytes with high expression of inducible nitric oxide synthase (NOS2) (41), which induces apoptosis of epithelial cells. Another mediator of influenza-associated lung injury is myeloperoxidase (MPO), which is predominantly secreted by neutrophils during cases of severely pathogenic influenza infections (42). Neutrophil Elastase (ELANE) is an inflammatory mediator of neutrophils that is predictive of development of acute lung injury (ALI) or acute respiratory distress syndrome (ARDS), however its role in influenza infections is debatable (43–46). TNF-related apoptosis-inducing ligand (TRAIL), released by NK cells and inflammatory monocytes, interacts with death receptors on the surface of epithelial cells to induce apoptosis (47). Given that Tpl2 ablation leads to increased recruitment of inflammatory monocytes and neutrophils to the lungs, we next assessed the lung expression of pro-inflammatory cytokines and chemokines that recruit these cells as well as their effector molecules that could potentially damage the pulmonary epithelium and compromise lung function. Consistent with protein data, we noted overexpression of various pro-inflammatory cytokine mRNAs in lung tissue from influenza-infected *Tpl2^-/-^* mice at 7 dpi, including IFN-β, IFN-γ, IL-6 and the anti-inflammatory cytokine IL-10, as well as overexpression of chemokines CCL2, CXCL1, CCL5 and CXCL10 which are collectively involved in recruitment of inflammatory monocytes and neutrophils (**Figures 4A–L**). We also examined the level of CXCL2, another neutrophil recruiting chemokine (48) known to be active in bacterial infection models and found no difference between WT and *Tpl2^-/-^* lung tissue (**Figure 4K**). On testing for various inflammatory mediators, including NOS2, MPO, ELANE and TNFRSF10 (the gene encoding TRAIL), we observed upregulation of only NOS2 in the lungs of *Tpl2^-/-^* mice at 7 dpi (**Figures 4M–P**), suggesting that elevated NOS2 secretion by the increased numbers of inflammatory monocytes or neutrophils may contribute to morbidity. Notably, we observe that the IFN-β mRNA expression correlated with CCL2 mRNA expression (**Figure 4S**), and NOS2 mRNA expression in the lungs of the *Tpl2^-/-^* mice correlated with both CCL2 and IFN-β expression (**Figures 4T, U**), supporting the hypothesis that over-expression of IFN-β in *Tpl2^-/-^* mice stimulates increased production of the IFN-inducible gene, CCL2, which is responsible for the recruitment of NOS2-expressing monocytes that contribute to the damage of the pulmonary epithelium. Additionally, CXCL1 mRNA expression positively correlated with both IFN-β and NOS2 mRNA expression in the lungs of *Tpl2^-/-^* mice (**Figures 4V, W**), even though CXCL1 was not upregulated by protein expression at 7 dpi.

Because IFN-γ protein and mRNA expression were very high in influenza-infected *Tpl2^-/-^* mice (**Figures 2C** and **4D**) and have been reported to induce damage *via* NOS2 (49), we also examined whether IFN-γ correlated with NOS2 mRNA expression and did not find any correlation (**Figure 4X**). To further address the source of the high levels of IFN-γ, *Tpl2^-/-^* mice and *Tpl2^-/-^Rag1^-/-^* mice were infected with influenza, and IFN-γ protein levels in lung homogenates were assessed at 7 dpi. Both *Tpl2^-/-^* and *Tpl2^-/-^*/*Rag^-/-^* mice that lack T cells, produced similar levels of IFN-γ protein (**Supplementary Figure 5A**), suggesting that T cells are not the source of IFN-γ overproduction. Collectively, these significant correlations support the hypothesis that the expression of NOS2 is linked to the recruitment of inflammatory monocytes and neutrophils under the influence of IFN-β overexpression and the most likely cause of the morbidity seen in the *Tpl2^-/-^* mice.

### Influenza-Infected Tpl2^-/-^ Mice Exhibit an Increased Interferon Response That Cannot Be Adequately Controlled by SOCS1-Mediated Regulation

**Figure 5:**
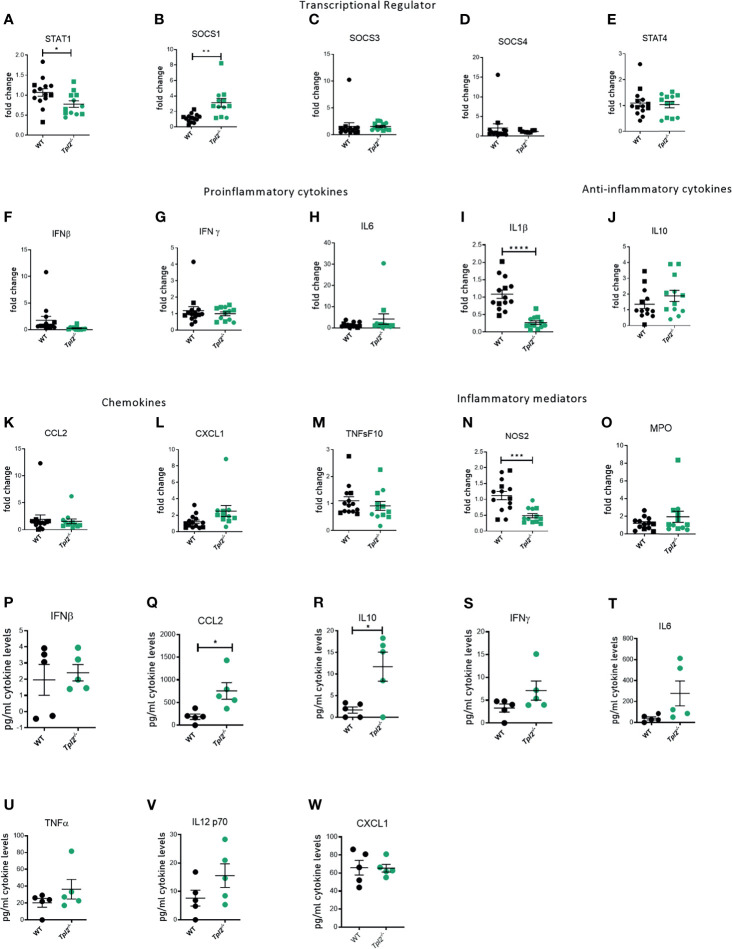
Ineffective suppression of CCL2 protein levels despite transcriptional repression in influenza-infected *Tpl2^-/-^* mice at 9 dpi. WT (n = 5) and *Tpl2^-/-^* (n = 5) mice were infected intranasally with 10^4^ pfu of influenza x31 and euthanized at 9 dpi. **(A–O)** WT (14) & *Tpl2^-/-^* (11) lungs were homogenized and analyzed for gene expression for transcriptional regulators **(A–E)**, pro-inflammatory cytokines **(F–I)**, anti-inflammatory cytokines **(J)**, chemokines **(K, L)** and inflammatory mediators **(M–O)**. **(P–W)** The lungs were homogenized and assessed for cytokine protein levels. Squares represent male mice, and circles represent female mice. Data are representative of 2 experiments. Unpaired student’s *t*-test *p<0.05, **p<0.01, ***p<0.001, ****p<0.0001.

The IFNs signal primarily *via* activation of the JAK/STAT pathway, with STAT1 playing a central role for both Type I and II interferons (50). Furthermore, interferons participate in a feed-forward loop with IFN-β amplifying the signal through Interferon Alpha Receptor 1 (IFNAR1) by inducing multiple IFNαs and other interferon-stimulated genes (ISGs), such as CCL2 (50, 51). Finally, resolution of this pathway is mediated in large part by the IFN-mediated induction of suppressors of cytokine signaling 1 (SOCS1), which downregulates interferon expression and signaling *via* STAT1 (52). Because *Tpl2^-/-^* mice show higher recruitment of inflammatory monocytes and neutrophils as the infection progresses, we hypothesized that Tpl2 either limits the amplitude or promotes resolution of the antiviral IFN response. To address the regulation of the IFN pathway by Tpl2 in response to influenza infection, we first measured the expression of both STAT1 and SOCS1, which serve as positive and negative regulators of the IFN pathway, respectively. At the peak of morbidity in *Tpl2^-/-^* mice at 9 dpi, STAT1 was markedly downregulated in the lungs of *Tpl2^-/-^* mice (**Figure 5A**), but not earlier at 7 dpi (**Figure 4Q**). In order to determine the cause of STAT1 downregulation, we assessed expression of the various SOCS genes and found that SOCS1 was overexpressed by 7 dpi (**Figure 4R**) and remained elevated through 9 dpi (**Figure 5B**). This upregulation was specific to SOCS1 and not seen with the other SOCS or STAT proteins (**Figures 5C–E**). Notably, the IFNs and ISGs were no longer upregulated at a transcriptional level in *Tpl2^-/-^* mice at 9 dpi, suggesting that elevated SOCS1 is suppressing the IFN response (**Figures 5F–P**). Additionally, in *Tpl2^-/-^* mice, SOCS1 is upregulated at 9 dpi while all IFNs and ISGs decline to WT levels by 9 dpi, providing further evidence of SOCS1-mediated transcriptional repression of the interferon response. Overexpression of SOCS1 in *Tpl2^-/-^* lung tissue was associated with increased levels of IL-10 protein in the lungs (**Figure 5R**), indicative of a reparative response. However, despite elevated SOCS1 and decreased NOS2 mRNA expression in *Tpl2^-/-^* mice at 9 dpi (**Figures 5B, N**), CCL2 protein levels remained elevated (**Figure 5Q**) and were accompanied by trending higher levels of other pro-inflammatory cytokines, including IFN-γ and IL-6 (**Figures 5S–V**). Cxcl1 levels were not different (**Figure 5W**), consistent with protein levels observed at 7 dpi (**Figure 2H**). Therefore, we conclude that inefficient regulation of the IFN/STAT1 pathway *via* SOCS1 permitted persistently elevated levels of CCL2, the chemokine recruitment signal for monocytes, in *Tpl2^-/-^* mice at the peak of morbidity (9 dpi). Collectively, these findings suggest that dysregulation of the IFN pathway in *Tpl2^-/-^* mice promotes excessive and prolonged influx of inflammatory cells that contribute to lung damage and morbidity observed in influenza-infected *Tpl2^-/-^* mice.

### In Chimeras With Tpl2 Ablation Restricted to Radioresistant Cells, Hypercytokinemia Is Suppressed

**Figure 6:**
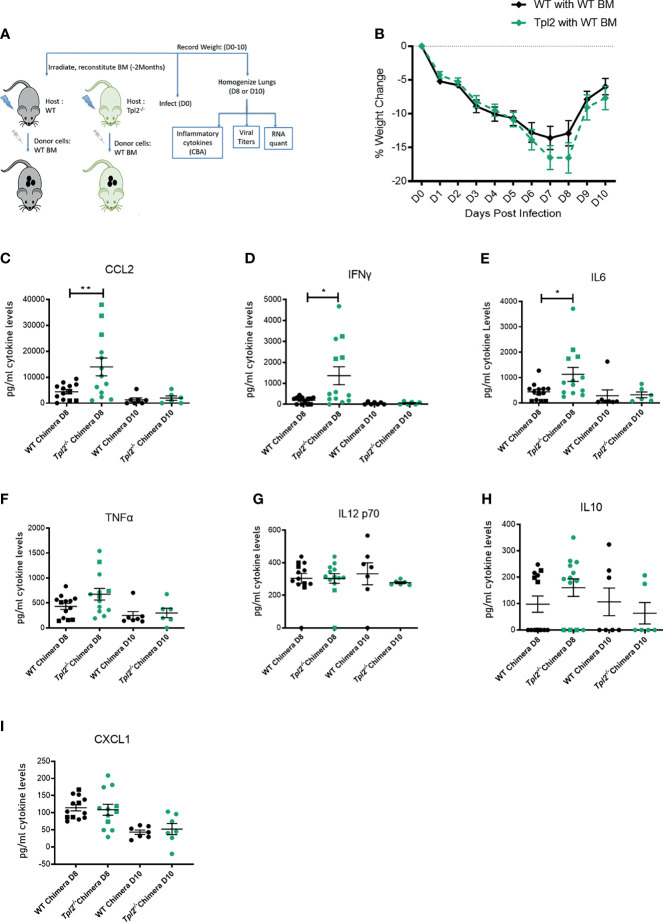
Tpl2 ablation in the radioresistant cells allows for an initial cytokine burst at 8 dpi, but chimeras recover by 10 dpi. **(A)** Experimental outline for infection of chimeras. WT or *Tpl2^-/-^* mice were irradiated and given WT bone marrow to reconstitute for 2 months. They were then infected and studied for 8-10 days for clinical outcome and cytokine examination on day 8 or day 10 post infection. **(B)** Weight loss curve shows that the *Tpl2^-/-^* chimeras show greater weight loss by day 8, but are able to recover their weights by day 10 post infection. Diamonds represent that the data points are averaged for males and females. **(C–I)** Cytokine expression at day 8 or day 10 post infection. Unpaired student’s t-test *p<0.05, **p<0.01.

In an effort to localize Tpl2 functions that regulate hypercytokinemia in late stages of influenza infection, we generated chimeras using WT or *Tpl2^-/-^* recipient mice that were given WT donor bone marrow post irradiation, ensuring that hematopoietic cells would be of WT origin post recovery (as outlined in **Figure 6A**). Differential weight loss was observed in the *Tpl2^-/-^* chimeras from 7 to 8 dpi (**Figure 6B**), after which the mice recovered. Upon examination of the cytokines in the lungs, CCL2, IFN-γ and IL-6 were all upregulated in *Tpl2^-/-^* chimeras (**Figures 6C–E**) at 8 dpi, whereas the levels of other pro-inflammatory cytokines such as TNF, IL-12, IL-10 and CXCL1 were not affected (**Figures 6F–I**). Upregulation of CCL2, IFN-γ and IL-6 at 8 dpi in *Tpl2^-/-^* chimeras suggests that the cytokine dysregulation is partially attributed to Tpl2 deficiency in radioresistant cells, such as epithelial (53, 54) or stromal cells (55). Importantly, the regulation of these cytokines normalized in *Tpl2^-/-^* chimeras by 10 dpi, at which time the Tpl2 chimeras fully recovered. This overall phenotype is unlike the prolonged cytokine dysregulation and progressive weight loss seen in germline *Tpl2^-/-^* mice at 9 dpi (**Figure 1A**), suggesting that *Tpl2^-/-^* chimera recovery is mediated by suppression of hypercytokinemia by WT hematopoietic cells. As alveolar macrophages are also part of the lung resident immune cell population and are radioresistant (56), we considered the possibility that the source of the dysregulated cytokines at 7 dpi was the alveolar macrophages rather than the epithelial or stromal cells. However, we found no differences in the gene expression for CCL2, IL-6, IFN-γ or IFN-β at 7 dpi in sorted alveolar macrophages from WT and *Tpl2^-/-^* lungs (**Supplementary Figures 6A–D**).

## Discussion

**Figure 7:**
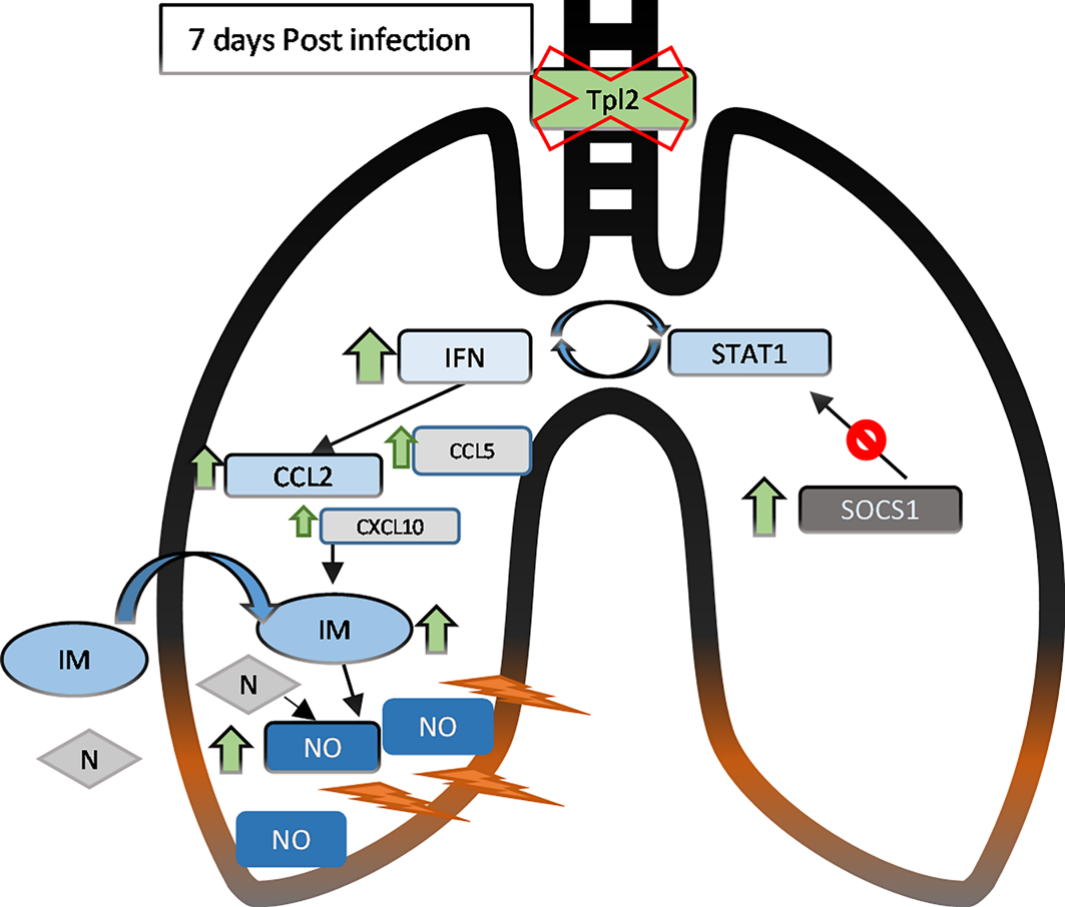
In the lungs of influenza-infected *Tpl2^-/-^* mice at 7 dpi, upregulation (green arrows) of the IFNs and chemokines leads to recruitment and retention of inflammatory monocytes (IM, ovals) and neutrophils (N, diamonds) that lead to lung damage likely *via* nitric oxide (NO). IFN signaling also activates the SOCS1 transcriptional repressor, that suppresses STAT1 mRNA levels to limit further IFN signaling by 9 dpi. However, inefficient SOCS1-mediated repression in *Tpl2^-/-^* mice allows persistent CCL2 overexpression and inflammation, progressing to morbidity and mortality.

*Tpl2^-/-^* mice exhibit enhanced morbidity and mortality to influenza infection with deteriorating clinical symptoms from 7 to 9 dpi. Live virus was undetectable by 9 dpi, confirming complete, albeit delayed, viral clearance in the *Tpl2^-/-^* mice as noted in our previous study (22). Despite viral clearance, the *Tpl2^-/-^* mice showed hypercytokinemia and influx of inflammatory cells, specifically inflammatory monocytes and neutrophils, by 7 dpi. Increased inflammatory monocyte and neutrophil recruitment in *Tpl2^-/-^* mice coincided with increased expression of type I interferons and the inflammatory mediator NOS2 (**Figure 7**). These findings demonstrate that Tpl2 serves a regulatory role during influenza infection by tempering the production of type I interferons and IFN-stimulated chemokines that leads to excessive recruitment of inflammatory cells known to cause physical trauma to the pulmonary epithelium (10, 17, 40, 57).

Infection of mice with virulent strains of influenza leads to increased expression of IFNs and concomitant overexpression of CCL2, which induces excessive recruitment of inflammatory monocytes and immunopathology (17, 40, 41, 58). Consistent with these studies, severe weight loss in *Tpl2^-/-^* mice was associated with increased expression IFN-β. High IFN-β expression correlated with increased CCL2 and NOS2 expression, supporting IFN-β-CCL2-NOS2 as an axis of monocyte-mediated recruitment and inflammation in *Tpl2^-/-^* mice. Importantly, IFN-β and CCL2 were also both overproduced at the protein level in influenza-infected *Tpl2^-/-^* mice, further supporting this mechanism of regulation. Although high CXCL1 correlated with elevated IFN-β and NOS2 at the mRNA level in *Tpl2^-/-^* mice, lack of CXCL1 protein overexpression at 7 dpi suggests translational control of this chemokine in the *Tpl2^-/-^* mice despite high mRNA levels, questioning the contribution of this pathway in cellular recruitment. Other chemokines elevated in influenza-infected *Tpl2^-/-^* mice, including CXCL10, CCL5, CCL3 and CCL4, are also overexpressed in human cases of lethal influenza infections (8, 11) and recognized for recruitment of inflammatory monocytes and neutrophils (10). Notably, CCL3 (MIP-1α) has also been recognized as a neutrophil recruiter (59), and CCL3 was increased in the *Tpl2^-/-^* at the protein level at 7 dpi (**Figure 2K**). Importantly, IL-6 and chemokines like CCL3, CCL4 and CCL5, which are not classically induced by IFNs, are also upregulated in *Tpl2^-/-^* mice, indicative of a generalized inflammatory response. However, overexpression of IFNs (IFN-β/IFN-γ) and IFN-inducible chemokines, like CCL2 (51, 58) and CXCL10 (60), suggest a more pronounced alteration of these IFN pathways. According to multiple lines of evidence (8, 9, 61), targeted dysregulation in these pathways is sufficient to cause the excessive recruitment of the inflammatory monocytes and neutrophils, consistent with the phenotype of influenza-infected *Tpl2^-/-^* mice at 7 dpi.

It is important to note that the excessive recruitment of monocytes and neutrophils in *Tpl2*^-/-^ mice reported herein during influenza infection was unexpected based upon the recruitment phenotypes observed in *Tpl2^-/-^* mice using other inflammatory models. Multiple studies have shown that Tpl2 ablation leads to *decreased* recruitment of both macrophages and neutrophils in response to inflammation induced by zymosan, acetaminophen, caerulein or thioglycollate administration (31, 38, 39, 62, 63). However, these studies have focused on the acute effects (within 72 hours) of Tpl2 ablation unlike the later phenotype assessed herein. In this regard, we previously noted similar levels of IFN-β at 1 and 3 dpi with influenza (22), consistent with similar cellular recruitment profiles at 4 dpi (**Figures 3I, J**), suggesting an important kinetic component. Another important distinction in the models is the differential expression of type I IFNs, which are known for their immunomodulatory effects. Influenza infections are characterized by high levels of type I IFNs compared to the acute inflammatory models used to assess innate immune cell recruitment in *Tpl2^-/-^* mice (31, 38, 39, 62, 63). Infection of *Tpl2^-/-^* mice with *Mycobacterium tuberculosis* results in a high type I IFN signature that impairs antibacterial functions *via* induction of IL-10, reminiscent of the present findings (21); however, potential effects of Tpl2 on pulmonary recruitment of monocytes and neutrophils was not assessed in this model (21). Collectively, these studies emphasize the importance of kinetic regulation of cytokines and chemokines in promoting inflammation. Furthermore, they suggest that stimuli that promote strong type I IFN responses are likely to elicit uncontrolled inflammation in *Tpl2^-/-^* mice.

The use of bone marrow chimeras revealed important information about the source of the Tpl2-dependent immunoregulation during influenza infection. We observe upregulation of the IFN response in later stages of the infection in germline *Tpl2^-/-^* mice as well as chimeras, although this was not sustained in the chimeras. Transient IFN overexpression resolved by 10 dpi, corresponding with complete recovery of *Tpl2^-/-^* chimeras. These findings indicate that Tpl2 ablation in radioresistant cells like the pulmonary epithelium or endothelium leads to an initial cytokine dysregulation and overexpression at 7 dpi. The full recovery of *Tpl2^-/-^* chimeras compared to the high morbidity of germline *Tpl2^-/-^* mice further suggests that Tpl2 also functions to some extent within the hematopoietic compartment to limit influenza-induced inflammation. Overall, these findings suggest that the source of hypercytokinemia is an interplay between Tpl2-dependent effects in both radioresistant stromal cells and radiosensitive hematopoietic cells.

The most prominent radioresistant lung cell populations that are susceptible to influenza infection are the Type 1 and Type 2 airway epithelial cells as well as alveolar macrophages. While the alveolar macrophages are susceptible to infection, they express lower levels of cytokines than peripheral blood monocyte derived macrophages (64). Furthermore, the similar expression levels of IFN-β and CCL2 (among others) by alveolar macrophages isolated from influenza-infected WT and *Tpl2^-/-^* mice suggest that dysregulated cytokine responses in influenza-infected *Tpl2^-/-^* mice likely originate from other cellular sources, like the pulmonary epithelial cells that are primary targets and replicative niches for influenza. Cell-type specific regulation of the type I interferons by Tpl2 has been characterized in multiple immune cell types, including macrophages, DCs and pDCs. However, evidence of Tpl2-dependent regulation of epithelial cell functions is sparse. One study of intestinal inflammation using the DSS model has demonstrated that Tpl2 is essential for intestinal homeostasis, with Tpl2-deficient mice showing extensive intestinal inflammation characterized by focal ulceration, loss of Goblet cells and loss of crypts (65). The protective role for Tpl2 in that study was shown to be intrinsic to intestinal myofibroblasts that sense epithelial damage and signal homeostatic responses *via* a Tpl2-COX-2-Prostaglandin E2 pathway. Another study demonstrated that Tpl2 signals ERK1/2 activation in response to *Pseudomonas* antigens and several purified TLR ligands *via* TAK1 and IKK-β in BEAS-2B immortalized human bronchial epithelial cells, and Tpl2 inhibitor treatment resulted in decreased *Pseudomonas*-induced IL-6 and IL-8 secretion (66). A follow-up study from the same group showed that Tpl2 also promoted IL-33 expression in response to *Pseudomonas aeruginosa via* the same pathway in airway epithelial cells expressing a Cystic Fibrosis mutation (CFTRdelF508) (24). Unfortunately, none of these studies provides insight into whether or how Tpl2 regulates type I interferon production in pulmonary epithelial cells. Ongoing studies are addressing the Tpl2-dependent regulation of antiviral responses specifically within pulmonary epithelial cells. However, the current findings suggest that overall Tpl2 functions as a negative regulator of type I IFNs late during influenza infection, which is consistent with the negative regulation observed in macrophages and dendritic cells (20, 22).

Antiviral IFNs are potent inhibitors of viral spread, but they also stimulate strong inflammatory responses, including antigen presentation by dendritic cells and T cell differentiation and activation, and their dysregulation can lead to immunopathologies (67–69). The necessarily tight control over IFN signaling is achieved, in part, by the actions of a family of eight SOCS proteins that inhibit JAK/STAT signaling. Not only did we observe overexpression of IFNs and ISGs such as STAT1, CCL2 and IFN-γ at 7 dpi, but we also observed a striking induction of SOCS1 in the lungs of the *Tpl2^-/-^* mice. SOCS1 is an ISG induced during influenza infection to inhibit the expression of IFNs and their downstream signaling by inhibiting STAT1 or JAK1, which are required for signaling *via* this pathway (52, 70). Moreover SOCS1 can be stimulated in response to influenza by a wide range of pathways including RIG-I, MAVS and the IFNAR1 pathway and can concomitantly downregulate other ISGs including STAT1, IFN-β and IRF-3 (52). SOCS3 is another ISG that has been found to downregulate similar ISGs as SOCS1 and impacts the regulation of IL-6 *via* the STAT3 pathway independent of the IFN signaling pathway (52, 71, 72). Additionally, *Socs4^-/-^* mice are highly susceptible to influenza infections due to elevations in key inflammatory cytokines such as IL-6, IFN-γ and CCL2 with impaired trafficking of virus specific CD8 T cells to the lungs, highlighting the importance of SOCS4-mediated regulation of the influenza response (73). Among the various SOCS family members, we observed consistently upregulated levels of SOCS1 in *Tpl2^-/-^* mice from 7 to 9 dpi. This increase in SOCS1 in *Tpl2^-/-^* mice presumably assisted in the downregulation of STAT1 and other ISGs such as NOS2 by 9 dpi. Despite the downregulation of CCL2 mRNA at 9 dpi, corresponding reductions at the protein level were delayed, and overexpression of CCL2 and IL-10 proteins were still evident at 9 dpi. Therefore, negative regulation of the interferon pathway is operative in the *Tpl2^-/-^* mice, although delayed such that CCL2 protein levels are inefficiently suppressed at 9 dpi, potentiating cellular infiltration in *Tpl2^-/-^* mice late during infection.

Nitric oxide synthase 2 (NOS2) is one of the inflammatory mediators shown to cause epithelial cell damage and thereby lead to morbidity in influenza-infected mice that present with hypercytokinemia (41, 57). NOS enzymes catalyze the production of nitric oxide (NO) from L-arginine. Notably, NOS and NO have previously been implicated in damage to the pulmonary epithelium. First, higher expression of NO has been observed in mice infected with highly pathogenic avian influenza strains compared to seasonal strains, and antibody blockade of NO led to increased survival (57). Second, *NOS2^-/-^* mice survived infection with a low pathogenicity virus strain *via* an IFN-γ-dependent anti-viral mechanism, demonstrating that NOS2 contributed more to influenza-mediated pneumonitis rather than viral control in WT mice (49). Although NO expression is not restricted to either inflammatory monocytes or neutrophils (57), NO expression by other sources such as the epithelium is low and transient (74). Similarly, NO expression by alveolar macrophages is also restricted, being stimulated by IFNγ only from macrophages that are in contact with type II alveolar epithelial cells (74, 75). Importantly, during influenza infection, the primary source of NOS2 has been demonstrated to be inflammatory monocytes (17, 41). NOS2 is also expressed to a lesser extent by neutrophils, which were also increased in *Tpl2^-/-^* mice (**Figure 3C**). However, failure to detect coincident elevations in MPO expression (**Figures 4O** and **5O**), a hallmark neutrophil effector molecule, in *Tpl2^-/-^* mice suggests that neutrophils are not a dominant mediator of pulmonary damage in this model or they are working in concert with inflammatory monoctyes (16, 76, 77). Therefore, it is likely that the numerically increased inflammatory monocyte pool, with additional neutrophil contribution in the *Tpl2^-/-^* mice induces lung pathology *via* their expression of NOS2 and NO.

Analyses of peripheral blood during influenza infection in humans has demonstrated upregulation of Tpl2 expression at days 4 and 6 post infection (78). We show that Tpl2 tempers severe immunopathology during influenza infection in mice *via* suppression of late-stage cytokine regulation. Future studies should examine the correlation of Tpl2 expression with influenza outcomes, as Tpl2 expression may represent a diagnostic tool in the prediction of severe immunopathology during influenza infection. Furthermore, a better understanding of immunoregulation of influenza infections by Tpl2 could also guide the discovery of immunotherapies for cases of hypercytokinemia.

## Data Availability Statement

The original contributions presented in the study are included in the article/**Supplementary Material**. Further inquiries can be directed to the corresponding author.

## Ethics Statement

The animal study was reviewed and approved by University of Georgia Institutional Animal Care and Use Committee.

## Author Contributions

KL and WW conceptualized and designed the experiments. KL and KJ performed the experiments and analyzed the data. KL and WW wrote the manuscript. All authors contributed to the article and approved the submitted version.

## Funding

Research reported in this publication was supported by the National Institute of Allergy and Infectious Diseases of the National Institutes of Health under Award Number R21AI147003-01 to WW. The content is solely the responsibility of the authors and does not necessarily represent the official views of the National Institutes of Health.

## Conflict of Interest

The authors declare that the research was conducted in the absence of any commercial or financial relationships that could be construed as a potential conflict of interest.

## Publisher’s Note

All claims expressed in this article are solely those of the authors and do not necessarily represent those of their affiliated organizations, or those of the publisher, the editors and the reviewers. Any product that may be evaluated in this article, or claim that may be made by its manufacturer, is not guaranteed or endorsed by the publisher.

## Abbreviations

Tpl2, Tumor progression locus 2; Dpi, days post infection; NOS2, nitric oxide synthase; IAV, influenza A virus; WT, Wild type; x31, Influenza virus A/HKx31; BALF, Bronchoalveolar lavage fluid; IFNAR1, Interferon Alpha Receptor 1; ISGs, interferon-stimulated genes; SOCS1, suppressors of cytokine signaling 1.
