# Supplementary material for: Tpl2 Ablation Leads to Hypercytokinemia and Excessive Cellular Infiltration to the Lungs During Late Stages of Influenza Infection
Source: Front Immunol. 2021 Oct 7;12:738490. doi: 10.3389/fimmu.2021.738490 (PMC8529111; doi:10.3389/fimmu.2021.738490)
Supplement: Supplementary file 1 [file DataSheet_1.pdf]

Supplemental Figure 1

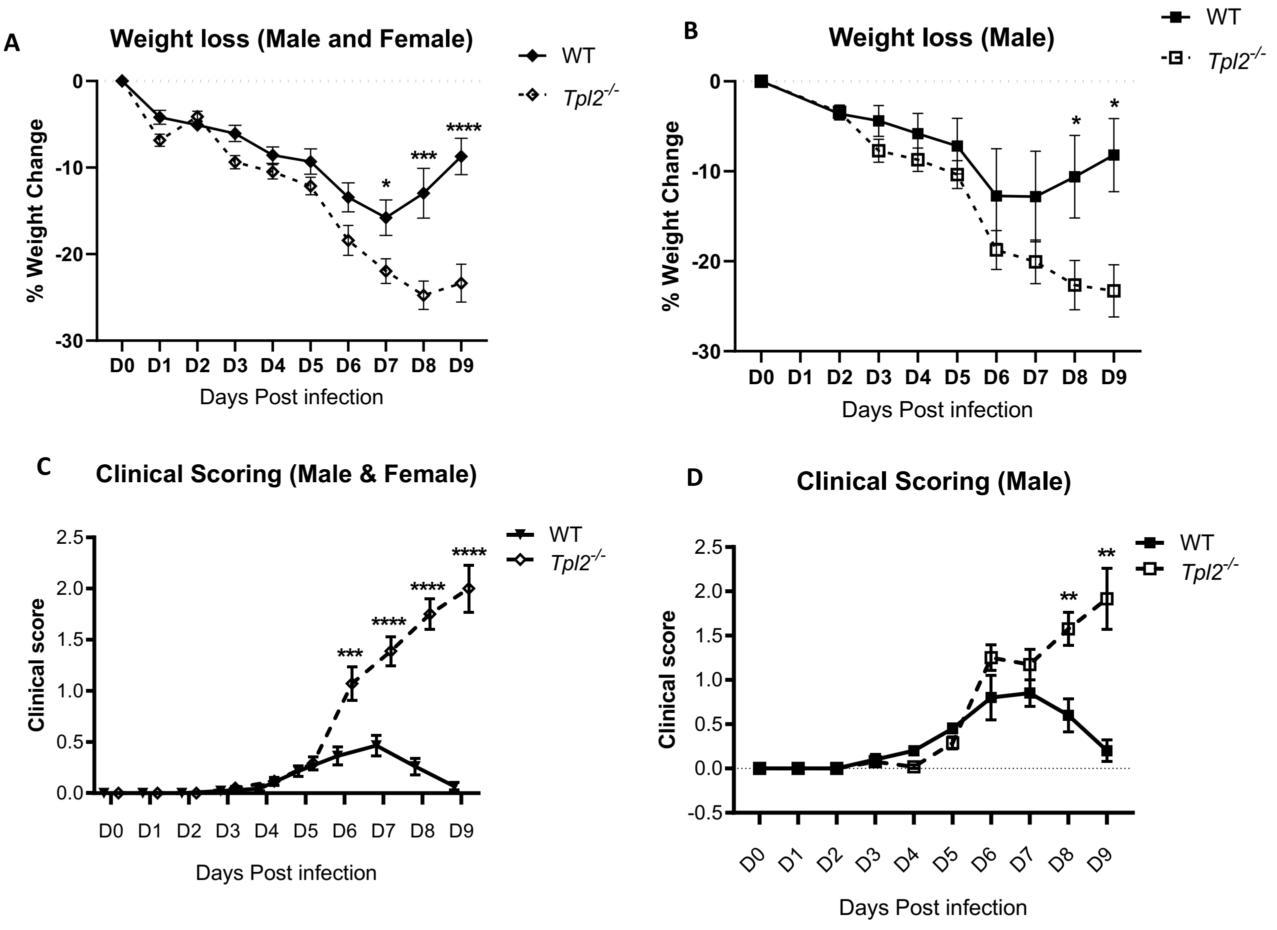

Supplementary Figure 1. Male mice show similar weight loss and clinical scores as female mice in response to influenza A virus. (A) Percent weight change of both female and male WT (n=21) versus *Tpl2*$^{-/-}$ (n=30) mice 9 dpi with 10^4 pfu influenza A virus strain x31. Data are representative of 5 experiments. Unpaired student's *t*-test; *p<0.05, **p<0.01, p<0.001, ****p<0.0001 (at each dpi). (B) Percent weight change of male WT (n=5) versus male *Tpl2*$^{-/-}$ (n=14) mice 9 dpi with 10^4 pfu influenza A virus strain x31. Data are representative of 3 experiments. (C-D) Progression of clinical symptoms including lethargy, piloerection, and hunching is shown throughout the course of infection for both sexes (C) with data representative of 5 experiments and for males only (D) with data representative of 3 experiments. Unpaired student's *t*-test; *p<0.05, **p<0.01, ***p<0.001, ****p<0.0001. Diamonds denote the inclusion of both males and female data points in the average; squares represent male data points only included in averages.

Supplemental Figure 2

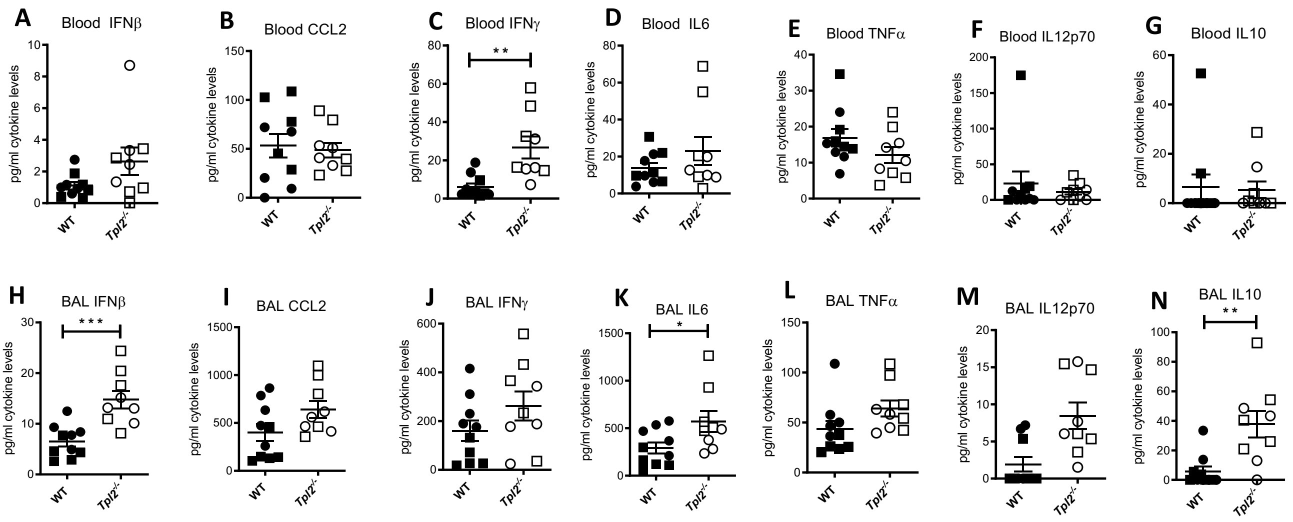

Supplementary Figure 2. Increased blood and BAL cytokines in influenza-infected *Tpl2$^{-/-}$* mice at 7 dpi. WT (n=10) and *Tpl2$^{-/-}$* (n=9) mice were infected intranasally with 10^4 pfu of influenza x31 and euthanized at 7 dpi. **(A-G)** Blood was collected by cardiac puncture to analyze the cytokine expression**. (H-N)** PBS was intratracheally injected into the broncho-alveolar spaces to collect the BAL fluid which was used to analyze cytokine expression. Squares represent male mice, and circles represent female mice. Data are representative of 2 experiments. Unpaired student's *t*-test. *p<0.05, **p<0.01, ***p<0.001.

Supplemental Figure 3

A

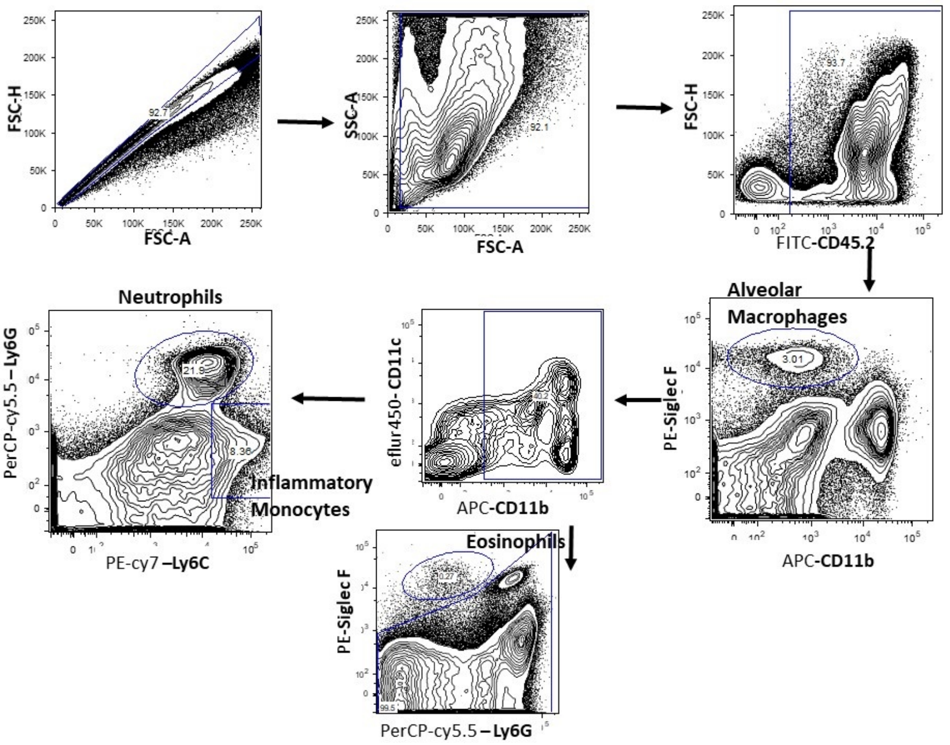

B

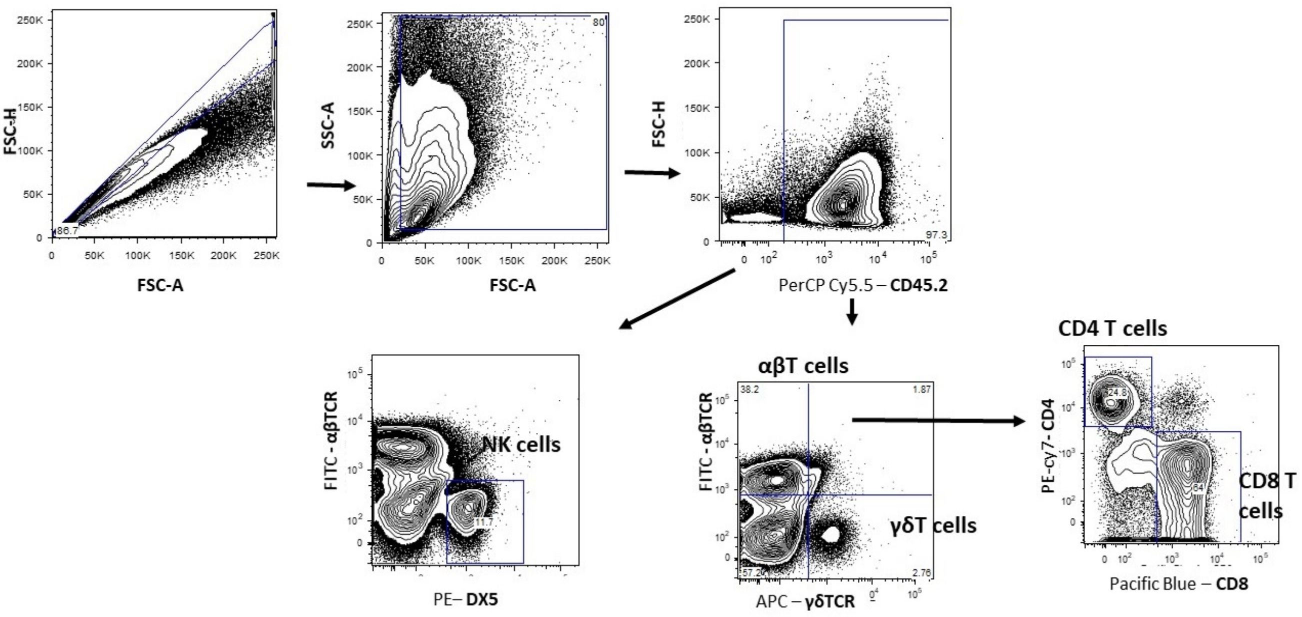

Supplementary Figure 3. Flow cytometry gating strategy used to differentiate populations. (A) Siglec F, CD11b, CD11c, Ly6C, Ly6G, CD45.2 (Stain 1); **(B)** TCRαβ, TCRγδ, CD4, CD8, DX5, CD45.2 (Stain 2)

Supplemental Figure 4

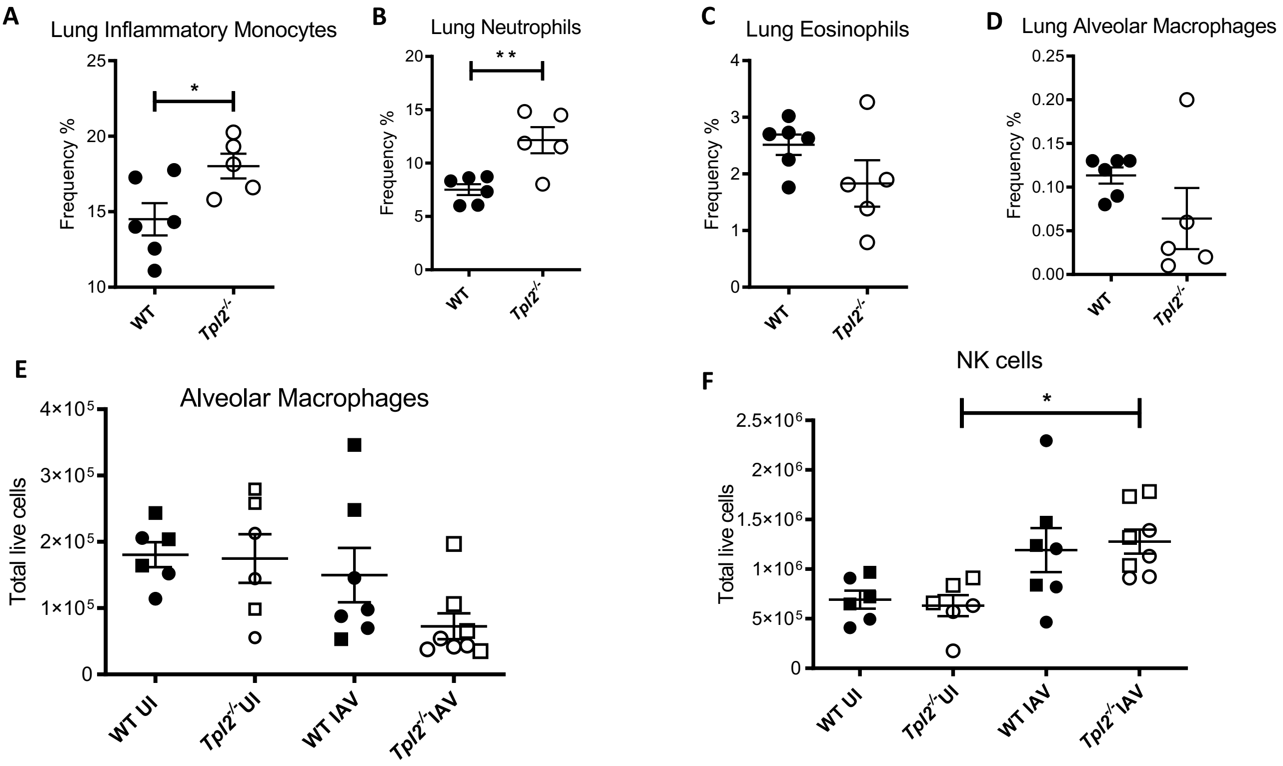

Supplementary Figure 4. Additional cellular profiling at 4 dpi and 7 dpi. WT (n=6) and *Tpl2$^{-/-}$* mice (n=5) were infected intranasally with 10^4 pfu of influenza x31 and euthanized at 7 dpi. The lungs were lavaged, perfused with PBS, digested with collagenase, and interstitial leukocytes were enriched by Percoll density gradient centrifugation. Squares represent male mice, and circles represent female mice. (A-D) lung cell frequencies (post lavage, perfusion and digest) are shown. (E-F) Total cell numbers in lung (post lavage, perfusion and digest) at 4 dpi were determined by flow cytometry as specified in *Materials and Methods*. Data are representative of 3 experiments. Unpaired student's *t*-test. *p<0.05, **p<0.01.

Supplemental Figure 5

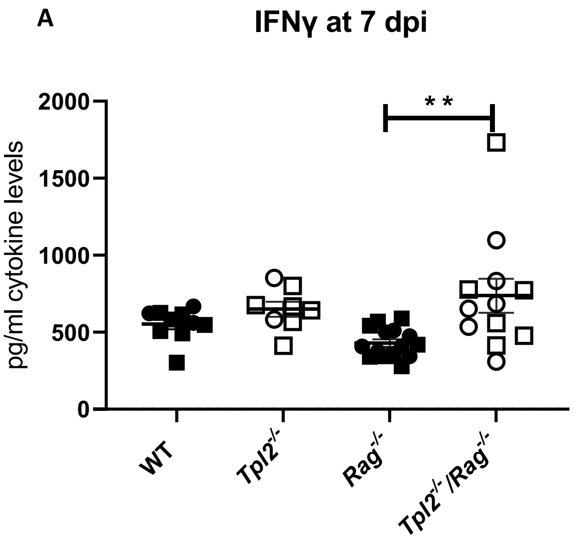

Supplementary Figure 5. Interferon-γ levels are not affected in the absence of T cells in *Tpl2$^{-/-}$/Rag$^{-/-}$* mice compared to *Tpl2$^{-/-}$* infected mice at 7 dpi. WT (n=10), *Tpl2$^{-/-}$* mice (n=8), *Rag$^{-/-}$* (n=15) and *Tpl2$^{-/-}$/Rag$^{-/-}$* (n=12) were infected intranasally with 10^4 pfu of influenza x31 and euthanized at 7 dpi. (A) IFNγ levels in the homogenized lungs (not perfused or lavaged) were assayed by Peprotech ELISA from infected mice at 7 dpi. Data are representative of two experiments. Squares represent male mice, and circles represent female mice. Unpaired student's *t*-test. *p<0.05, **p<0.01.

Supplemental Figure 6

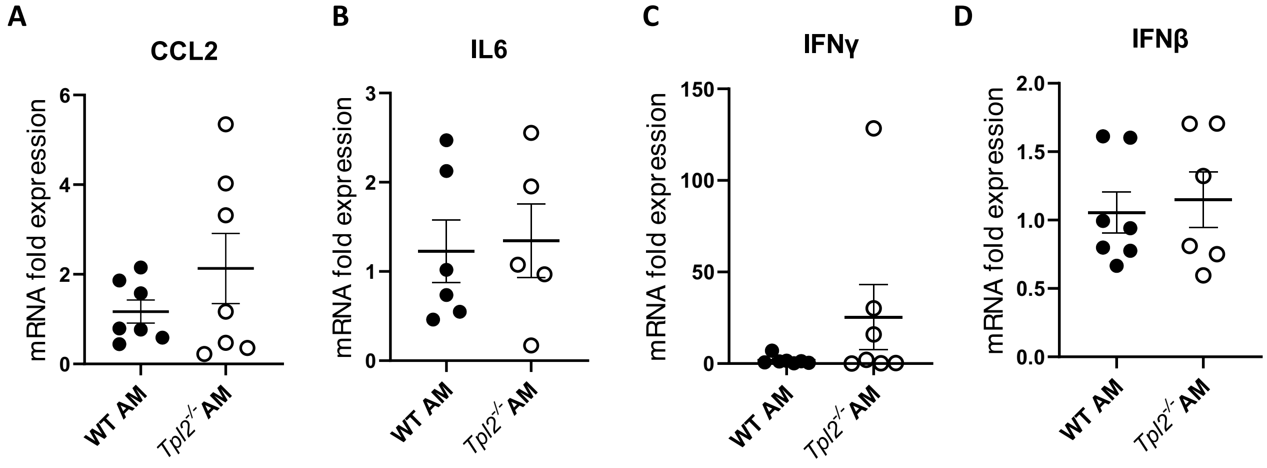

Supplementary Figure 6. No difference in the levels of proinflammatory mediators in WT and *Tpl2*$^{-/-}$ alveolar macrophages. WT (n=7) and *Tpl2*$^{-/-}$ (n=7) mice were infected intranasally with 10^4 pfu of influenza x31 and euthanized at 7 dpi. Their lungs were digested with collagenase, and alveolar macrophages (AM) were sort purified based on the flow gating strategy in Supplementary Figure 3. (A-D) The cells were lysed in TRK lysis buffer, and the RNA was extracted, converted to cDNA and analyzed by RT-PCR relative to WT AM, which was designated a value of 1. Data are representative of two experiments. Unpaired student's *t*-test was used to compare between WT and *Tpl2*$^{-/-}$. Female mice were used for these experiments.
